# Supplementary figures and images for: Heterologous Expression Screens in Nicotiana benthamiana Identify a Candidate Effector of the Wheat Yellow Rust Pathogen that Associates with Processing Bodies
Source: PLoS One. 2016 Feb 10;11(2):e0149035. doi: 10.1371/journal.pone.0149035 (PMC4749346; doi:10.1371/journal.pone.0149035)

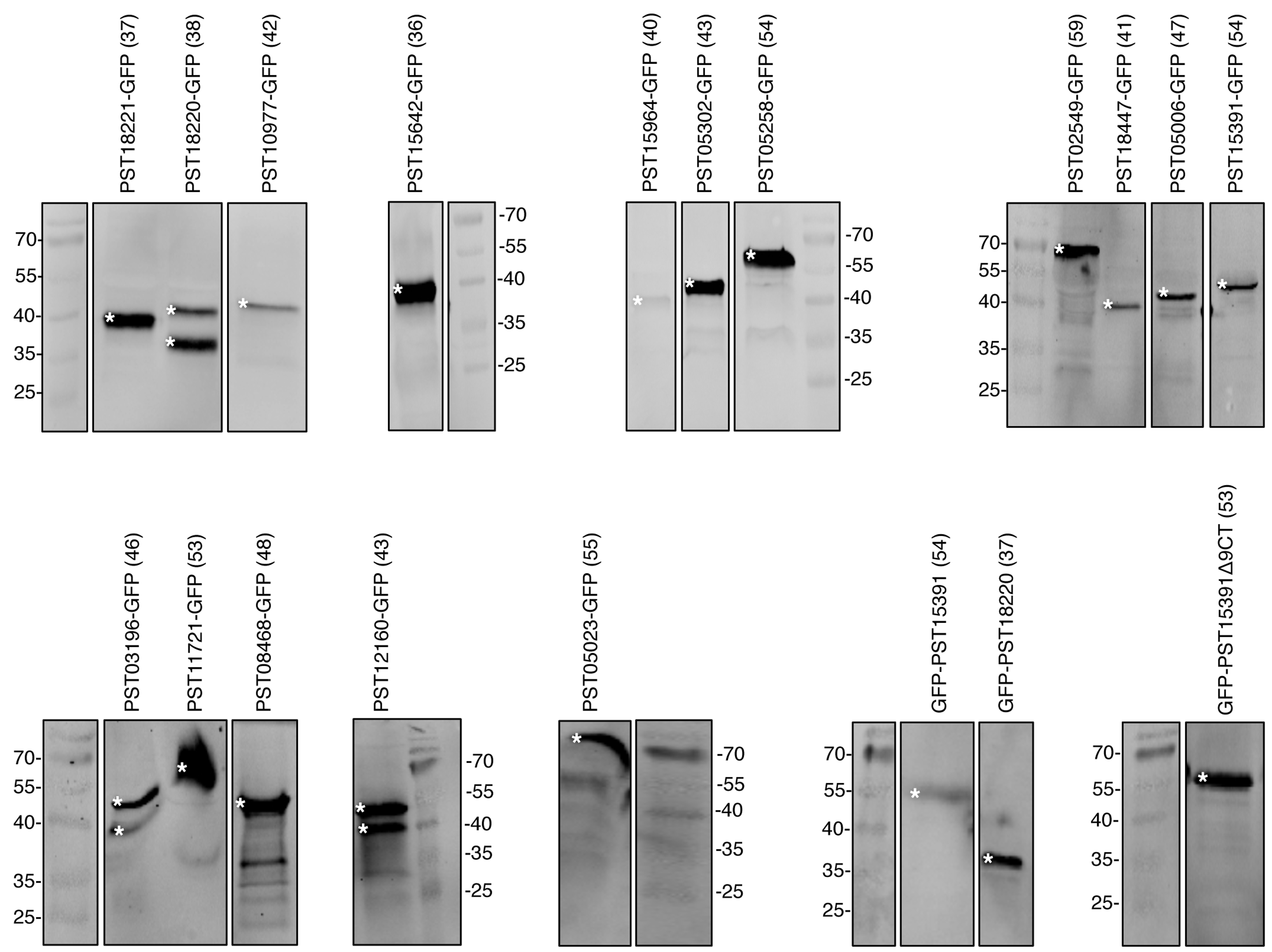

Supplement: S1 Fig — Proteins were transiently expressed in N. benthamiana leaf cells by agroinfiltration. Total proteins were extracted two days after infiltration by grinding leaves in liquid nitrogen and immediately extracting, reducing and denaturing proteins from the leaf powder. Proteins were separated on 15% SDS-PAGE gels and transferred onto a nitrocellulose membrane. Primary and secondary immune detection were performed with rabbit anti-GFP and goat anti-rabbit antibodies, respectively. Images originating from the same membrane and processed at the same time are grouped together. Blots were cropped to remove lanes previously published elsewhere (Petre et al., 2015a). Secondary antibodies and PageRuler signals were detected simultaneously using an infrared imager. The theoretical size of each fusion protein is indicated in kDa in parentheses. White asterisks indicate specific protein bands. (TIF) [file pone.0149035.s001.tif]

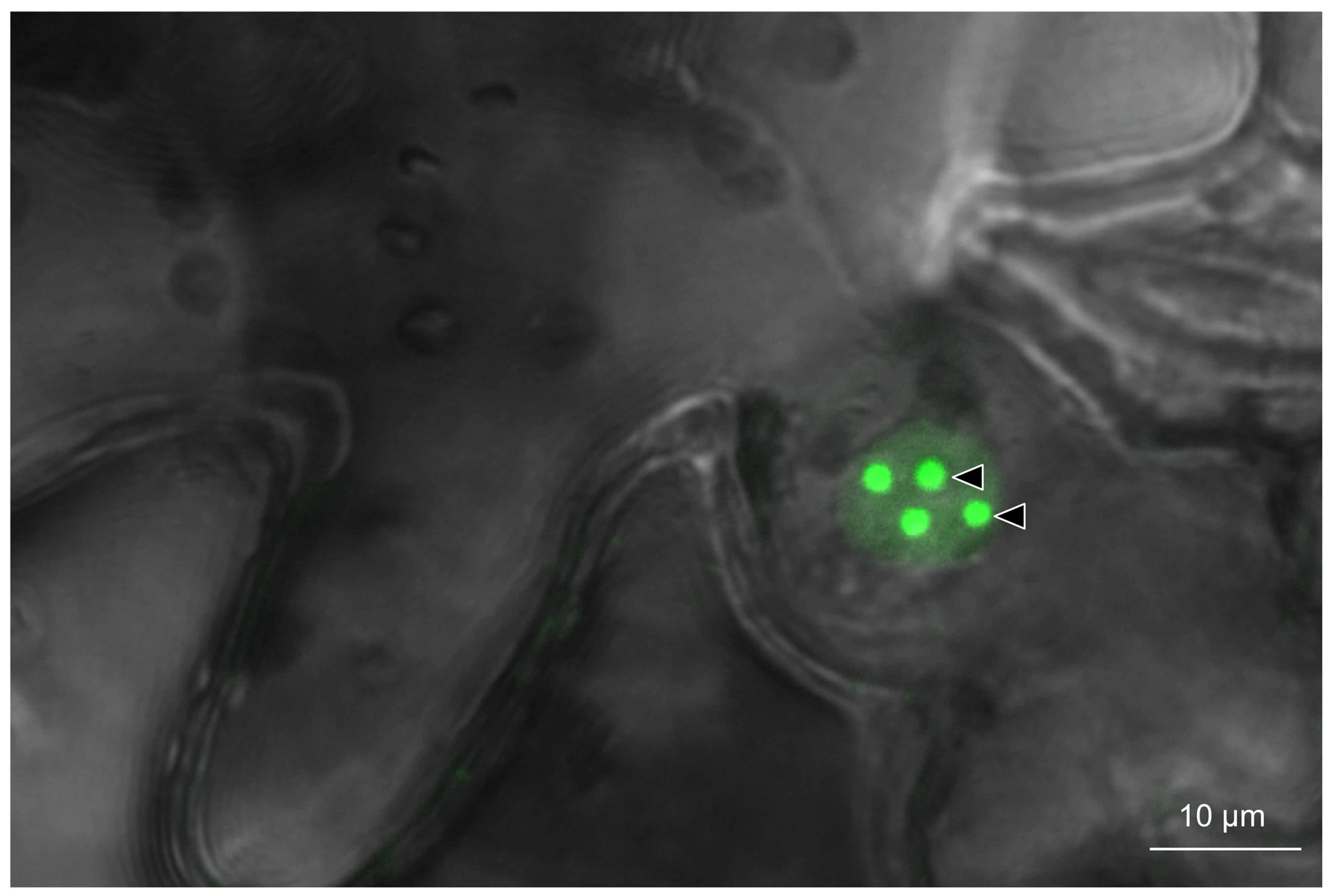

Supplement: S2 Fig — Live-cell imaging of PST11721-GFP in N. benthamiana leaf cells. Proteins were transiently expressed in N. benthamiana leaf cells by agroinfiltration. Live-cell imaging was performed with a laser-scanning confocal microscope two days after infiltration. The GFP was excited at 488 nm. GFP (green) fluorescence was collected at 505–550 nm. The image is a single optical section of 0.8 μm, showing an overlay of the GFP and bright field channels. The black arrowheads indicate GFP-labelled nuclear foci. (TIF) [file pone.0149035.s002.tif]

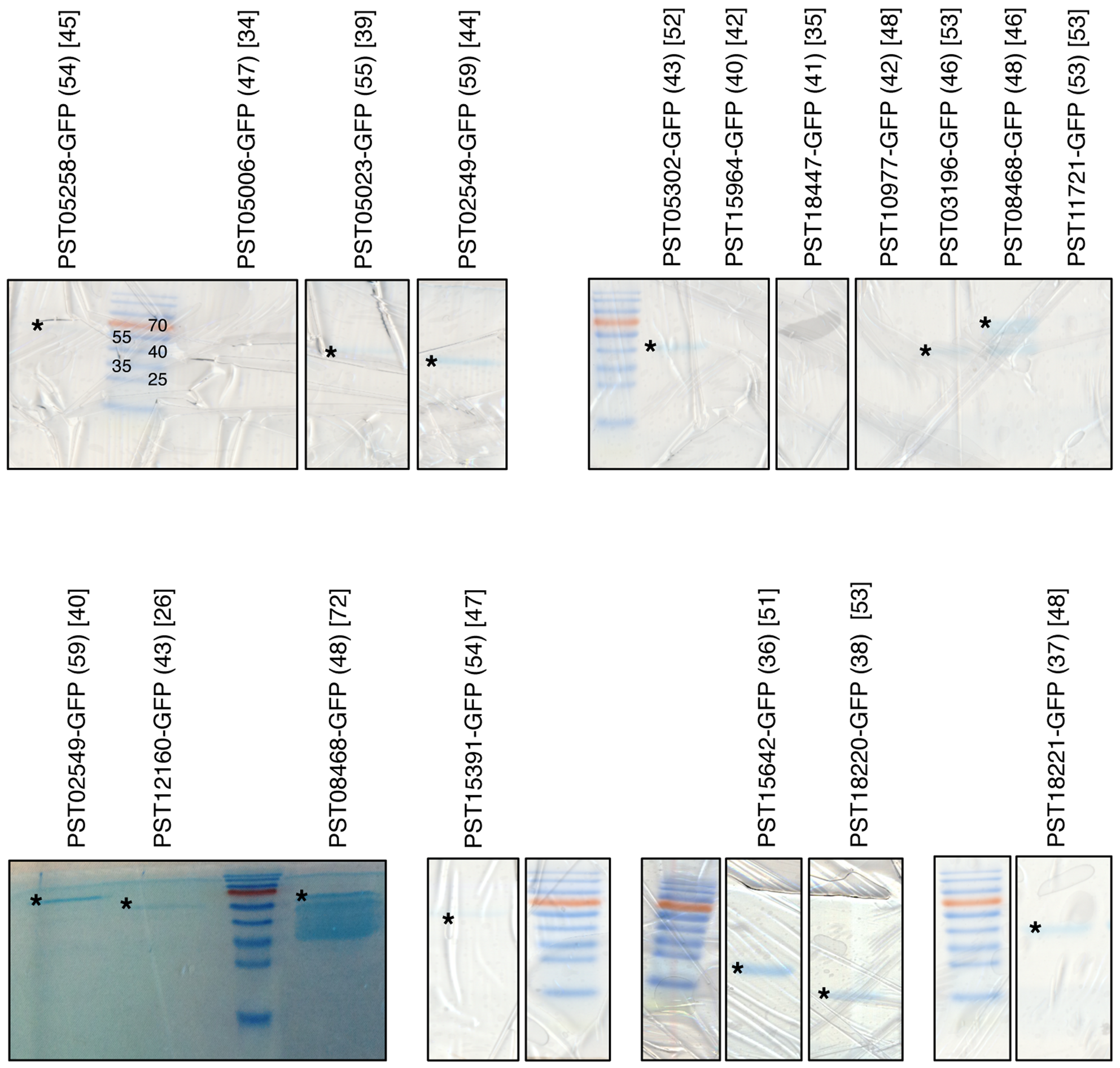

Supplement: S3 Fig — Protein mixtures isolated by anti-GFP immunoprecipitation were reduced and denatured in a Laemmli buffer. Proteins were separated with SDS-PAGE and stained with Coomassie Brilliant Blue. Trypsin-digested peptides were processed by LC-MS/MS and collected peaks were used to search a database containing the GFP sequence. The theoretical size of each fusion protein is indicated in parentheses in kilodalton (kDa). The number of peptides identified by LC-MS/MS and matching the GFP is indicated for each fusion protein between brackets. The size of the PageRuler ladder bands is indicated in kDa. Images originating from the same gel are grouped together. Black asterisks indicate detectable and specific protein bands. (TIF) [file pone.0149035.s003.tif]

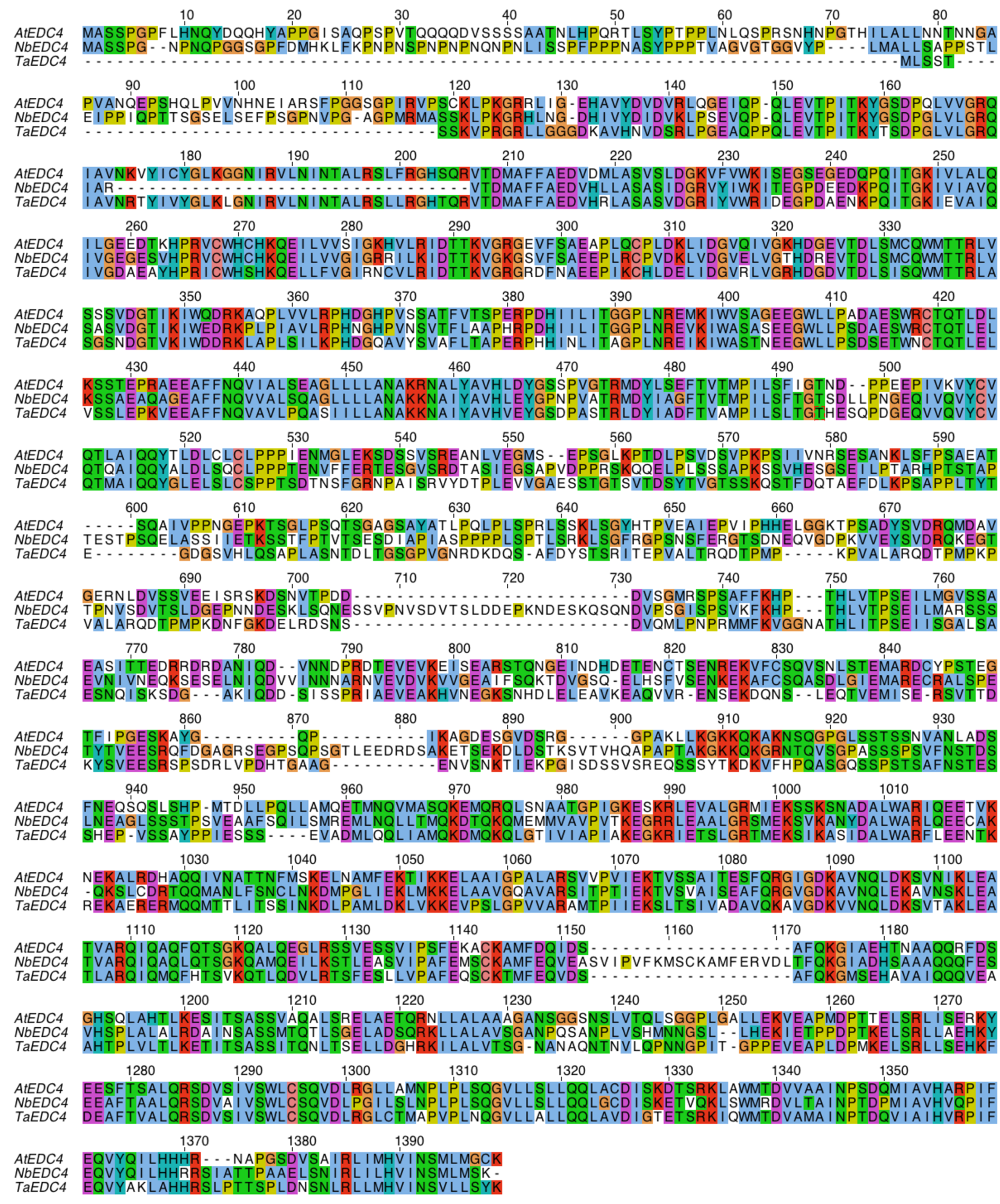

Supplement: S4 Fig — Amino acid alignment of EDC4 of Arabidopsis thaliana (AtEDC4, AT3G13300.2), Nicotiana benthamiana (NbEDC4, NbS00023257g0003.1), and Triticum aestivum (TaEDC4, Traes_6DL_3FBA5B70E.1). Alignment was performed with ClustalX. Amino acid residues are colored according to the ClustalX scheme. (TIF) [file pone.0149035.s004.tif]
